# Neonatal upper limb fractures – a narrative overview of the literature

**DOI:** 10.1186/s12887-024-04538-z

**Published:** 2024-01-19

**Authors:** Marcos Carvalho, Maria Inês Barreto, João Cabral, Inês Balacó, Cristina Alves

**Affiliations:** Department of Pediatric Orthopaedics, Pediatric Hospital of Coimbra - Centro Hospitalar e Universitário de Coimbra (CHUC), EPE; Av Afonso Romão, 3000-602 Coimbra, Portugal

**Keywords:** Neonatal upper limb fractures, Clavicle fracture, Humerus fracture

## Abstract

The aim of this paper is to review the topic of neonatal fractures of the upper limb, describing the different types of fractures focusing on the etiology, epidemiology, risk factors, clinical approach, diagnosis, treatment and prognosis of these injuries. We included all types of research studies, both experimental and observational, published in English, French, Portuguese and Spanish. The information was obtained using the keywords neonatal upper limb fracture, clavicle fracture or humerus fracture from the following resources: MEDLINE database, Embase^®^ database and LILACS database. Other resources such as hand searches of the references of retrieved literature and authoritative texts, personal and hospital libraries searching for texts on upper limb neonatal fractures, discussions with experts in the field of upper limb neonatal fractures and personal experience, were also considered for the completion of the article.

Neonatal fractures of the upper limb are consensually considered to have a good prognosis and no long-term sequelae. Conservative treatment is the option in the vast majority of the fractures and is associated with excellent results, with good healing, full range of motion, adequate remodeling without obvious deformity, neurologic impairment or functional implications.

## Background

Neonatal birth injury is defined as the impairment of neonatal body function or structure due to an adverse traumatic event that occur during labor, delivery or both [1, 2]. Reported risk factors for these injuries are advanced maternal age, maternal pelvic anomalies, abnormal presentation, gestational diabetes, abnormal labor, macrosomia, low birth weight, shoulder dystocia, route of delivery, use of instrumental delivery, emergency cesarean delivery and experience of labor teams [3–20].

Neonatal fractures are included in this group of injuries and can occur during the period of labor, delivery and immediate post-partum (particularly in neonates who need resuscitation in the delivery room). These fractures have an incidence of 2.9/1000 live births and can occur secondary to conditions of the mother, the fetus, or external to both [10]. The risk factors described for these type of fractures are: fetal macrosomia (>4000g), low birth weight (LBW) (<2500g), maternal obesity (BMI>40Kg/m2), gestational diabetes, maternal short stature, maternal pelvic anomaly, vaginal delivery with breech presentation, emergency cesarean delivery, shoulder dystocia, instrumented delivery (forceps delivery, vacuum extraction) and less experience of the delivery teams [3–32]. These fractures are classically associated with instrumented vaginal deliveries, although they can be observed in spontaneous eutocic deliveries or cesarean sections.

Some neonates, requiring direct admission to neonatal intensive care unit (NICU) from birth, also have some increased risk factors for limb fractures, namely: prematurity, LBW, malnutrition, birth injuries, trauma due to medical intervention or side effects of medication required for the compensation of their underlying pathology [33]. Though the overall rate of appendicular fractures diagnosed in NICU-hospitalized patients in some studies is less than 1%, some of these are only diagnosed subacutely and may even go undiagnosed, especially as these patients are more vulnerable and less mobile. In patients admitted to NICU, with no evident fracture mechanism, there seems to be 2 distinct injury profiles: fractures in patients requiring steroids, diuretics, nutritional supplements and ventilatory support and others associated with "recent bed procedures" [34].

Often, in some of these patients with neonatal fracture, it is difficult to impute a direct causal event to the fracture, often attributing it generically to neonatal osteopenia (transient neonatal osteoporosis) [22, 33, 35]. Osteopenia of premature infants is the result of decreased bone synthesis and increased bone resorption, which may be caused by systemic involvement associated with prematurity, malnutrition or lack of mechanical stimulation [35]. The possibility of metabolic and bone-fragility diseases should also be considered, since that frequently secondary osteopenia may lead to the occurrence of fractures, especially in preterm, LBW and chronic disease patients [36, 37]. In this context there are different clinical cases that report neonatal humerus and femur shaft fractures and highlight the importance of bearing in mind the possibility of the presence of diseases such as osteogenesis imperfecta, rickets or Vitamin D deficiency [38–41].

The evaluation of these patients, due to the complexity of the metabolic cause underlying the fracture, should be performed and treated within a multidisciplinary approach, involving different specialties and experiences in several areas such as neonatology, endocrinology, metabolic diseases, genetics, nutrition and orthopaedics [34].

Despite this, the majority of neonatal fractures are associated with risk factors which are important to know in order to predict and potentially avoid the occurrence of injuries and also to anticipate the parents' expectations, thus allowing for a more effective emotional management and a lower psychological impact towards the adverse event. The aim of this narrative review is to investigate this important topic, describing the different types of neonatal upper limb fractures with a focus on the etiology, epidemiology, risk factors, clinical approach, diagnosis, treatment and prognosis of these injuries.

## Methods

We present a narrative overview of the literature focused on upper limb neonatal fractures. We included observational and retrospective studies obtained from the following sources considering the period available online for each database.

- MEDLINE database, Embase^®^ database and LILACS database. The following keywords were chosen for the research: neonatal upper limb fracture, clavicle fracture or humerus fracture. Based on the obtained articles and their bibliographic references, articles were selected. Articles written in English, French, Portuguese and Spanish have been included. No language restrictions have been applied.
- Hand searches of the references of retrieved literature and authoritative texts.
- Personal and hospital libraries searching for texts on upper limb neonatal fractures.
- Discussions with experts in the field of upper limb neonatal fractures.
- Personal experience.

### Epidemiology, etiology and risk factors

Clavicle fracture is the most frequent birth related fracture with an incidence of 0.5-11.2/1000 live births [9–14, 32, 42, 43] while the humerus fracture is the second most common of the long bones with an incidence of 0.04-0.2/1000 live births [19, 22, 25, 44, 45].

There are several risk factors described for these injuries: advanced maternal age, maternal overweight or obesity, short stature, increasing birth weight, malpresentation, Type 1 diabetes, gestational diabetes, shoulder dystocia, vacuum delivery, use of oxytocin (need for labor – high risk pregnancies) and pain relief during labor [1, 9, 10, 12–14]. Of the risk factors described, macrosomia seems to be present in 20-50% of fetuses [9, 10, 13–15]. It’s also interesting to see that high birth weight is an important feature closely related with other risk factors such as pre-pregnancy obesity, diabetes, induction of labor, vacuum-assisted delivery and shoulder dystocia [17, 25, 32, 46–54]. Shoulder dystocia is also an important risk factor for humerus fracture and like high birth weight, may also increase the likelihood of concomitant brachial plexus injuries [11, 43, 50, 55]. In patients with perinatal brachial plexus injury, studies report humerus fracture rates ranging from 2 to 11% [56, 57].

The association with instrumented vaginal deliveries is frequent in the literature and the majority of these are performed due to maternal distress or suspected fetal asphyxia or distress [11, 34]. Högberg states that assisted vaginal delivery by vacuum extraction was associated with clavicle fracture in 21.8% of the cases, while shoulder dystocia was present in only 4.3% of clavicle fractures [10]. The most frequent mechanism of injury during delivery occurs when maneuvers are performed to reduce the chest circumference in shoulder dystocia or by compression of the fetal anterior shoulder of the fetus being compressed against the maternal pubic symphysis leading to fracture [10]. Nevertheless, Kekki in his study states that although rare, there is an incidence of 0.46/1000 live births of clavicle fractures associated with cesarean sections, 66% of which occurred in unplanned procedures [11]. This association is also observed in fractures of the humerus, which although historically associated with breech maneuvers during vaginal delivery, there has been a relevant increase associated to caesarean deliveries that accompanies the growing popularity of this technique. Although caesarean deliveries avoid the risk of head entrapment when the baby is in breech position, the breech maneuvers necessary to extract the baby are similar to those performed in vaginal deliveries and may lead to fractures of the long bones [19, 58]. Despite of all this risk factors, approximately one quarter of the patients with clavicle fracture have no identified risk factor [11]. According to Madsen, within humeral fractures, transverse midshaft are the most common, followed by proximal humeral fractures and distal physeal fractures [22, 45]. Regarding epiphyseal separations of the distal humerus, these are very rare and are defined as epiphyseal fractures (Salter-Harris I or II), for which we must have a high diagnostic suspicion, since they often go undetected [19, 22, 45, 59].

### Clinical presentation

The clinical presentation of upper limb fractures in neonates is variable according to the fracture pattern and deviation. Displaced fractures are usually identified immediately after delivery due to their greater clinical exuberance. Local deformity, crepitus, oedema, pseudoparalysis of the affected limb and crying aroused by passive mobility of the limb may be present. Non-displaced fractures may only be diagnosed at a later stage (days or weeks), when local swelling due to bone callus formation is perceptible upon inspection or palpation. In the case of humerus fracture the Moro reflex is consistently asymmetric, whereas in non-displaced clavicle fracture there may not be an obvious asymmetry. Fracture of the clavicle is usually located in the middle third, while fracture of the humerus also occurs most frequently in the middle third, followed by the proximal third. Regarding the distal third of the humerus, this should be given special attention, as even after the clinical examination and initial complementary diagnostic study with X-rays, the diagnosis of tranphyseal fracture is not always unequivocal, justifying high suspicion index and other complementary means. A careful inspection of the whole limb is mandatory. The fracture pattern is usually simple (not comminuted) and with a transverse or oblique fracture line. Due to the possibility of associated perinatal brachial plexus injury mainly to clavicle and proximal humerus fracture, it is also important to document the motor function of the elbow, wrist and hand (spontaneous and after stimulation) and to maintain surveillance after the expected period of consolidation, in order to assess the evolution of the active mobility and function of the affected limb (Table 1).

**Table 1:** Red flags in neonatal upper limb fractures

	Red Flags	Suspect of	Tips
Neonatal Upper Limb Fractures	▪ Pseudoparalysis of the limb that does not resolve after 2-3 weeks	▪ Possible associated perinatal brachial plexus injury	- Document the motor function of the shoulder, elbow, wrist and hand (spontaneous and after stimulation) on day 1 - Pseudoparalysis due to humerus fracture delays the diagnosis of neurologic injury. The presence of active shoulder abduction, elbow flexion and grasp reflex, along with the absence of active wrist and finger extension, are typical findings of radial nerve palsy.
	▪ Drop wrist on clinical examination	▪ Possible associated radial nerve palsy	

### Diagnosis

**Fig. 1:**
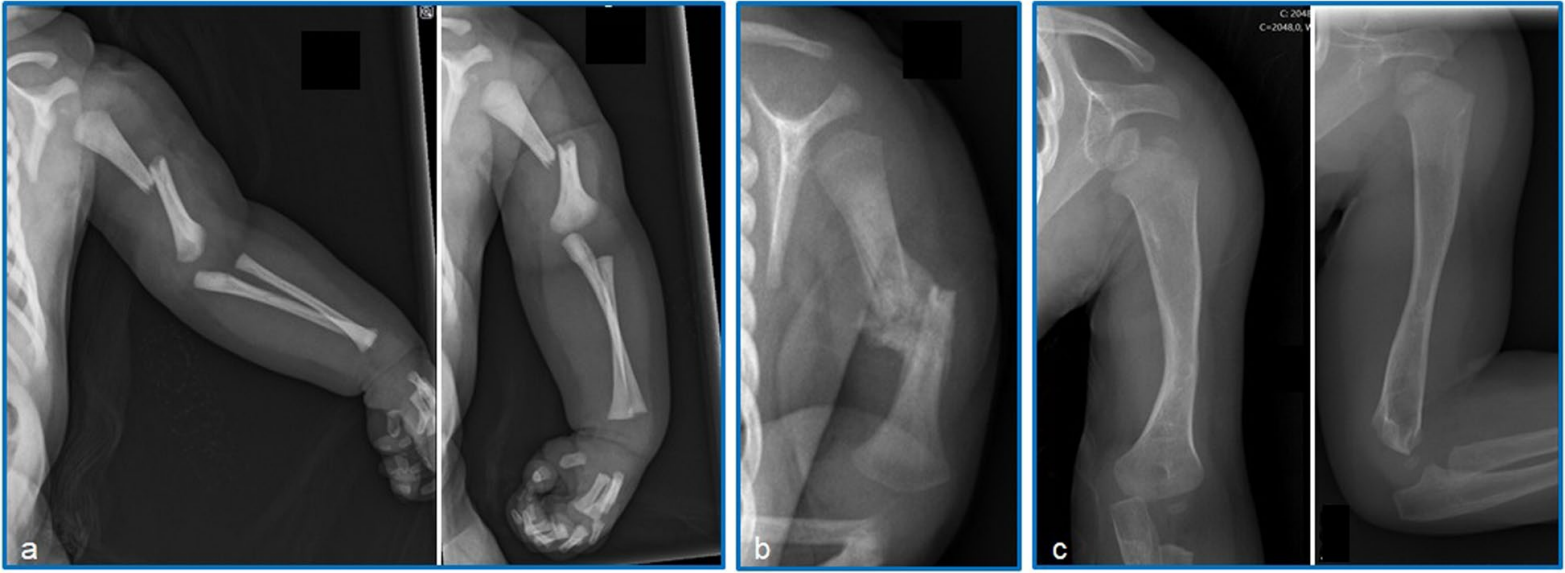
Male neonate, macrosomic (4024g), shoulder dystocia and instrumental delivery, with a right humeral shaft fracture, diagnosed on the 1st day of life, after limb deformity and humerus crepitus noticed immediately after delivery. Treatment was carried out with soft immobilization, placing the affected limb with the sleeve of a long-sleeved shirt attached to the chest with the elbow at 90º flexion. **a** X-ray on day 1 showing a right middle shaft humerus fracture with significant displacement. **b** At 3 weeks follow-up: X-ray with evidence of bone callus and indication for passive and active mobility of the right upper limb. **c** At 10 months follow-up: the patient was clinically without symptoms or restrictions and the X-ray shows clear evidence of consolidation and remodeling of the fracture

**Fig. 2:**
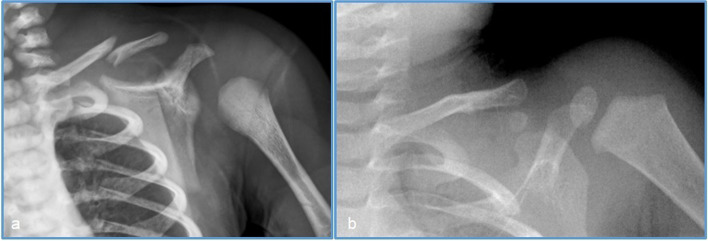
Male neonate, instrumented delivery, with a left clavicle fracture, diagnosed on the second day of life after perception of decreased mobility of the upper limb and crepitus of the clavicle. **a** X-ray on day 2 showing a middle third left clavicle fracture with deviation. **b** At 10 months follow-up: patient clinically without symptoms or restrictions and the X-ray shows clear evidence of consolidation and remodeling of the fracture

When in the presence of a clinical suspicion of fracture, the diagnosis of neonatal humeral diaphyseal or clavicle fracture is usually simple and almost always made by means of a radiograph, allowing differential diagnosis with other birth-related traumatic pathologies, such as perinatal brachial plexus injury or glenohumeral dislocation (Figs. 1 and 2) [60, 61]. Clavicle and humerus fractures may be associated with other traumatic bone or neurological injuries, thus the radiographic study should be amplified and not only focused on the suspected location of the fracture (consider requesting a chest and full upper limb X-ray). Neonatal humeral shaft fractures may present with neuropraxia of the radial nerve, an injury that, although usually belated diagnosed, should be identified and distinguished from perinatal brachial plexus injury. In these injuries, patients do not show active extension of the wrist or fingers but preserve an active abduction of the shoulder and a flexion and extension of the elbow that is similar to the contra-lateral side, although this is often only fully identified after immobilization is removed and resolution of the pseudoparalysis of the limb by the fracture (Table 2). This differential diagnosis is important as these cases have a good prognosis with 72% of patients making a full recovery after 2 months and 100% after 6 months [62, 63]. In addition, closed injuries of the radial nerve and median nerve in children are generally associated with favorable spontaneous recoveries, while lesions of the ulnar nerve are associated with a worse prognosis and have a more uncertain prognosis [64]. For this reason, in the presence of a radial nerve injury in the context of a humeral shaft fracture, the posture to adopt should be very conservative and expectant, since a complete resolution of the clinical picture is to be expected, in a self-limiting manner.

**Table 2:** Pitfalls and Pearls in Neonatal Upper Limb Fractures

	Radiographic Pitfalls	Pearls	Tips
Neonatal Upper Limb Fractures	▪ Apparent shoulder dislocation	▪ Suspect of a physeal fracture of the proximal humerus	- Do not attempt a joint reduction without clarifying the diagnosis. - Ultrasound is of great usefulness in establishing the diagnosis. If not available, arthrogram or MRI are possible options.
	▪ Apparent elbow dislocation	▪ Suspect of a distal transphyseal humerus fracture	

With this concept in mind most of the birth-related fractures are diagnosed on the first day of life or in the early neonatal period (between day 1 and day 7). Högberg in his study states that only 5.8% of clavicle fractures are diagnosed after this period (4.7% between day 8 and 28, and 1.1% after day 28) [10]. Still it is known that some fractures are undiagnosed, and there are several cases of fractures that are diagnosed incidentally by X-ray [9]. It is also important to note that transphyseal fractures of the distal humerus may be particularly difficult to identify and therefore justify a more cautious approach by requesting other complementary means of diagnosis, whenever there is suspicion of fracture [34]. In these cases the diagnosis is usually late, sometimes only made after accidental observation of callus formation on the X-ray or palpation of a rigid mass in the elbow region [45, 61, 65]. This results from the fact that the distal and proximal physis of the humerus are not yet ossified at birth and are therefore not clearly visualized in the X-rays [22, 45, 66, 67]. Jacobsen reinforces this idea, stating that most of his patients with distal humeral transphyseal injury were diagnosed between 9 and 30 days of life [59].

Furthermore, these injuries as well as proximal humeral physeal fractures, when observed by inexperienced doctors, are often confused with joint dislocations, and may lead to inadequate and inconsequent attempts of joint reduction [22, 65, 68]. For this reason, it is necessary to keep these entities in mind and carry out other complementary studies such as ultrasound, MRI or even arthrography, in some specific cases (Table 3) [65, 69–90]. In his article, Sherr-Lurrie mentions that of the 5 patients with distal or proximal epiphyseal fracture of the humerus identified, these injuries were not diagnosed by X-ray, but by ultrasound and in one of the cases MRI was even necessary for further clarification, in a fracture of the proximal physis of the humerus [22].

**Table 3:** Summary of the literature published in the last 15 years on patients treated for transphyseal distal humerus fracture sustained at birth

Author	Case(s)	Delivery	Age at Diagnosis (days)	Imaging	Treatment	Follow-up (months)	Complications/Remarks/Follow-up
Jacobsen [59] (2009)	6	Protrusion of arm	12	XR, arthrogram	Cast (no reduction)	16	- Callus on initial radiographs - Normal alignment
		Normal	2	XR,US	Traction + closed reduction + cast	78	- Normal alignment
		Breech twin arm stuck	14	XR	Cast (no reduction)	60	- Callus on initial radiographs - Normal alignment - Slightly reduced ROM
		Cephalic fast delivery	9	XR,US	Cast (no reduction)	54	- Callus on initial radiographs - Normal alignment
		Long	1	XR,US,MRI	Closed reduction + cast	21	- Slightly reduced valgus
		Normal	30	XR	Cast (no reduction)	120	- Callus on initial radiographs - Normal alignment
Söyüncü [81] (2009)	1	VD	5	XR,US,MRI	Open reduction + K-wire + posterior splint	16	- Complete ROM; Normal alignment
Catena [75] (2009)	1	CS	1	XR,US	Closed Reduction + cast	12	- Complete ROM; Normal alignment
Sherr-Lurie [22] (2011)	2	CS: n=1 VD: n=1	-	XR,US	Closed Reduction + cast	6	- Complete ROM; Normal alignment
Sabat [79] (2011)	1	CS	5	XR,MRI	Closed reduction + posterior plaster slab	1	- Complete ROM; Normal alignment
Navallas [80] (2013)	1	cs	1	XR,US	Closed Reduction + cast	4	- Complete ROM - 10º of varus
Kamaci [82] (2014)	1	CS	2	-	Closed reduction + percutaneus K-wire + long arm splint	6	- Complete ROM; Normal alignment
Patil [91] (2015)	1	VD	2	XR,US	Closed reduction + percutaneus K-wire + immobilization	2.5	- Complete ROM
Lin [83] (2016)	1	CS	5	XR,MRI, Arthrogram	Open reduction + K-wire + cast	2	- Complete ROM; Normal alignment
Tharakan [84] (2016)	1	VD		XR,MRI, Arthrogram	Closed reduction + percutaneus K-wire + posterior plaster splint	12	- Complete ROM; Normal alignment
Kay [65] (2017)	4	CS	1	XR	Immobilization	-	Normal alignment
			4	XR, Arthrogram	Closed reduction + percutaneus K-wire + immobilization	-	Normal alignment
			7	XR,US	Immobilization	-	Normal alignment
			1	XR, MRI	Immobilization	-	Normal alignment
Gigante [76] (2017)	5	VD	3	XR	Closed Reduction + cast	60	Complete ROM
		VD	1	XR,US	Closed reduction + percutaneus K-wire + cast	15	Complete ROM
		VD	2	XR	Closed Reduction + cast	27	Complete ROM
		CS	2	XR,US	Closed Reduction + cast	36	Complete ROM
		VD	1	XR	Closed Reduction + cast	12	5º of cubitus varus
Hariharan [87] (2019)	9	CS: n=6 VD: n=3	-	-	Surgery	-	-
Tan [85] (2022)	1	-	4	XR,US,MRI, Arthrogram	Closed reduction + percutaneus K-wire + cast	-	-
Galeotti [86] (2023)	10	CS: n=1 VD: n=9	1	XR	Closed reduction + cast	120	-Loss of reduction – 2^nd^ treatment: Closed reduction + cast - Complete ROM - Normal alignment - episodes of 5^th^ finger paresthesia
			8	XR,US,MRI	Closed reduction + cast	120	- Complete ROM; Normal alignment - elbow pain (occasional)
			3	XR,US,MRI	Closed reduction + cast	12	- Complete ROM - Normal alignment
			2	XR,US	Closed reduction + cast	15	-Loss of reduction - Second treatment: Closed reduction + percutaneus K-wire - Complete ROM - Normal alignment
			6	XR,US, Arthrogram	Closed reduction + cast	16	- Complete ROM - Normal alignment
			6	XR,US, Arthrogram	Closed reduction + percutaneus K-wire	24	- Complete ROM - Normal alignment
			9	XR,US, Arthrogram	Closed reduction + percutaneus K-wire	21	- Complete ROM - Normal alignment
			9	XR,US, Arthrogram	Closed reduction + percutaneus K-wire	16	- Complete ROM - Normal alignment
			3	XR,US, Arthrogram	Closed reduction + percutaneus K-wire	14	- Complete ROM - Normal alignment
			2	XR,US, Arthrogram	Closed reduction + percutaneus K-wire	12	- Complete ROM - Normal alignment

*CS* cesarean section, *FU* follow-up, *M* months, *ROM* range of motion, *US* ultrasound, *MRI* magnetic resonance imaging, *VD* vaginal delivery, *W* weeks, *XR* X-Ray

Ultrasound, although operator-dependent, is an excellent option as it is readily accessible, non-invasive, radiation-free, inexpensive and does not require sedation or general anaesthesia, unlike MRI or arthrography [22, 65, 69, 70, 78, 91]. These advantages are also particularly important in the context of patients hospitalised in NICUs where the fragility of their condition or the fact that they are less comfortable to mobilise makes the portability and non-invasiveness of the ultrasound even more relevant [22]. Ultrasound can also be used for the diagnosis of humeral shaft or clavicle fractures and is a diagnostic alternative that should be taken into account for different types of fractures. One thing to be pointed out is that this examination implies an anatomical pressure on the injured segment and a painful stimulus for the baby and that this situation also disturbs the execution and quality of the exam and therefore it must be done by experienced doctors who reduce as much as possible the time the child is in pain caused by the ultrasound probe. Whenever available, a high-resolution ultrasound should be used [71, 72].

### Treatment

Neonatal fractures of the humerus and clavicle are usually treated conservatively. There are different options, and immobilization is usually done using clothing, traction, splints or casts [19, 22, 45, 61, 66]. Reduction maneuvers are not usually necessary, although in cases of humeral fractures with severe displacement, it is possible to realign the fracture during immobilization [22].

One option for greater comfort for the baby, and to limit the mobility of the affected limb, involves a soft immobilization, wearing a long-sleeved shirt and placing the affected limb with the sleeve attached to the chest with the elbow at 90º flexion. This immobilization, although less relevant in cases of clavicle fracture, it is of greater interest in mid-shaft humerus fractures due to the more significant prevention of secondary rotational displacement. Sherr-Lurie reports a similar approach for proximal humerus fractures (simple swaddling) while for the fractures of the shaft and distal humerus, the preferred method was a closed gentle manipulation and an above-elbow plaster cast for 2 weeks, with the upper limb held against the body by the baby's shirt [22]. The author also states that neonates do not show any apparent discomfort with the immobilization performed [22].

Nevertheless it is likely that in the first 7 days, the patient may feel more discomfort/pain, in which case oral or rectal analgesic medication (paracetamol) may be administered. The presence of a visible and painless swelling around 7-10 days usually indicates the presence of an adequate consolidation process, without the need for additional X-rays or greater exposure to ionizing radiation. The beginning of active mobility of the limb appears around 2-3 weeks, corresponding to bone healing [22, 87]. Parents are instructed of the natural evolution and it is explained to them that they should take care of a gentle mobilization when it is necessary to dress or wash their children.

If the expected evolution is not observed, the radiographs may be repeated at around 4 weeks and a high index of suspicion should be maintained for other associated lesions, such as perinatal brachial plexus injury. Neurovascular injuries due to neonate humerus diaphyseal fractures are rare [92].

Parents should be warned that although the X-rays may show angular displacement, this is not expected to cause any functional impairment of the limb or lead to any future limitation, as it is expected that the child's growth will allow for complete bone remodelling and realignment.

In some cases of distal transphyseal fractures of the humerus, particularly displaced and unstable ones, surgical treatment options have been described, preferably by closed reduction and percutaneous fixation with K-wires [20, 34, 87, 91, 93].

### Prognosis

Neonatal fractures of the upper limb have a good prognosis and no long-term sequelae are expected. Non-operative treatment (short-term splinting or immobilization or observation) is the option in the vast majority of the fractures and is associated with excellent results, with good healing, full range of motion, adequate remodeling without obvious deformity, neurologic impairment or functional implications [19, 22, 45, 59, 61].

In specific and rare cases, such as transphyseal fractures of the humerus, the complications described in the literature include reduced range of motion, cubitus varus/valgus or the need for secondary surgical procedures. Transphyseal fractures of the distal humerus are those that can most commonly associate these coronal deviations, as the distal physis is the one that contributes least to humeral growth and one of those with the least capacity for remodelling in the whole skeleton [59, 65, 76, 87]. Within these complications, decreased range of motion and the cubitus varus are the most frequent [65, 76, 80, 87].

Nevertheless, the literature mentions that even in neonatal transphyseal fractures and regardless of the treatment option, the prognosis is good and the long-term results (although scarcely reported) are equally favourable [59, 65, 75, 76, 93, 94].

## Conclusions

Neonatal fractures are a poorly studied topic but one whose knowledge should be deepened, being relevant to the activity of different specialities (obstetricians, neonatologists, paediatricians and orthopaedic surgeons) who deal with the newborn and its potential risk factors for fracture. These fractures may be the first sign of other more serious metabolic or systemic diseases, so their identification is crucial in the primary approach to the newborn. In patients without other underlying pathologies, these fractures mostly have a favourable evolution and no future functional impact, but when undiagnosed they can potentially lead to late sequelae and functional limitation. The majority of these fractures occur in newborns with risk factors where maternal adverse factors stand out, but also the anthropometric characteristics of the baby and complications associated with childbirth are important factors to be valued. Identifying the diagnosis and implementing treatment early, can improve prognosis and minimise future morbidity, which is why it is crucial to increase awareness of this issue. In the future, it will be important to carry out further studies to clarify reliable predictors that will allow the implementation of preventive measures, anticipating fracture.

## Data Availability

The datasets used and/or analysed during the current study available from the corresponding author on reasonable request.
